# A two-center pilot study on the effects of clinical ethics support on coercive measures in psychiatry

**DOI:** 10.1186/s12888-022-04024-9

**Published:** 2022-06-01

**Authors:** Julia Stoll, Anna Lisa Westermair, Ulrike Kübler, Thomas Reisch, Katja Cattapan, René Bridler, Robert Maier, Manuel Trachsel

**Affiliations:** 1 Institute of Biomedical Ethics and History of Medicine, University of Zurich, Zurich, Switzerland; 2 Clinical Ethics Unit, University Hospital Basel and University Psychiatric Clinics Basel, Basel, Switzerland; 3 Department of Psychosomatic Medicine, University Hospital and University of Basel, Basel, Switzerland; 4 Sanatorium Kilchberg, Zurich, Switzerland; 5 Psychiatriezentrum Münsingen, Münsingen, Switzerland; 6 University Hospital of Psychiatry and Psychotherapy, University of Bern, Bern, Switzerland

**Keywords:** Clinical ethics support services, Clinical ethics consultation, Moral case deliberation, Coercion, Seclusion, Mechanical restraint, Coerced medication, Psychiatry, Ethics, Quality of care

## Abstract

**Background:**

The use of formal coercion such as seclusion, mechanical restraint, and forced medication is one of the most challenging and complex issues in mental health care, on the clinical, the legal, and the ethical level. Clinical ethics support aims at assisting healthcare practitioners in determining the morally most justifiable course of action in these situations. However, the effectiveness of clinical ethics support has hardly been studied so far.

**Methods:**

Monthly moral case deliberation (MCD) was implemented in two acute wards of two different psychiatric hospitals in Switzerland. Frequency and intensity of coercion was measured on ward level (*n*_patients_ = 405), and the Moral Attentiveness Scale, Knowledge on Coercion Scale, and Staff Attitudes towards Coercion Scale were applied on healthcare practitioner level (*n*_HP_ = 46). Pre-post-comparisons were conducted using multi-level modeling where appropriate.

**Results:**

After implementation of MCD, formal coercion was less frequent (particularly seclusion, small effect size; 9.6 vs. 16.7%, *p* = .034, Cramér’s *V* = .105) and less intense (particularly mechanical restraint, large effect size; 86.8 ± 45.3 vs. 14.5 ± 12.1 h, exact *p* = .019, *r* = -.74), and approval for coercive measures among healthcare practitioners was lower when controlling for the number of MCD sessions attended.

**Conclusions:**

Clinical ethics support such as MCD may be a hitherto underutilized service for the reduction of coercion, complementing existing strategies and programs. Implementing clinical ethics support may help improve quality of care for persons suffering from severe mental illness.

**Supplementary Information:**

The online version contains supplementary material available at 10.1186/s12888-022-04024-9.

## Background

The use of coercion is one of the most challenging and complex issues in mental health care, on the clinical, the legal and the ethical level. Relative to other medical specialties, formal coercion is often applied in psychiatry [1]. *Coercion* can be defined as “measures carried out against the patient’s self-determined wishes or in spite of opposition” [2]. Coercion can be *formal* (use of force that is regulated by the law, such as involuntary commitment and seclusion) or *informal* (use of “softer” pressures such as persuasion, interpersonal leverage, inducement, and threat) [3, 4].

All types of coercion pose clinical and ethical challenges. While coercion is typically intended to benefit the patient by preventing self-harm and/or improving mental health, evidence for its effectiveness is limited [5, 6]. In addition, coercion generally has a negative psychological impact on patients (such as feelings of powerlessness, humiliation, and dehumanization) and can result in posttraumatic stress disorder (PTSD) [7, 6]. These challenges are compounded by difficulties in the assessment of decision-making capacity and the need to protect others from violent behavior, among others [7].

The extent of coercion varies widely across institutions and countries [8–13] and is associated with characteristics not only of the patients, but also the hospitals and wards (such as quality of leadership, policies, and staffing) and healthcare professionals^1^ (HPs; such as level of training and experience, and attitudes) [14, 8, 15]. It has also been suggested, but not yet examined, that HPs’ normative attitudes towards coercion, in turn, are influenced by their personal values [16].

This patient-independent variation in the extent of coercion indicates some potential for reducing coercion and thus improving quality of mental health care. HPs considering the use of coercion can be assisted in determining the morally most justifiable course of action through clinical ethics support (CES) [17]. The many different forms of CES include ethics consultations [18], ethics rounds [19], and moral case deliberation (MCD) [20]. In MCD, HPs meet to reflect collaboratively and systematically on a concrete clinical case. Commonly taking about 60 min, each MCD session is structured by one of several conversation methods, chosen according to the purpose of the session [20]. Methods can focus on the process (e.g., self-reflection, teambuilding, skills training) or the product (e.g., solutions, compromises, answers). Instead of giving normative recommendations, a trained MCD facilitator focusses on the quality of the deliberation process and the meaningfulness of the moral issues [20]. As MCD does not require a professional medical ethicist, implementing it might be less resource-intensive compared to other forms of CES, especially for non-tertiary care centers.

To date, there is limited evidence for the effectiveness of MCD in particular and CES in general [21], especially in mental health care [17]. While the existing evidence shows positive effects of CES, the outcome variables mostly focus on the HPs (and not on the patients and their relatives) and are subjective in nature, such as user satisfaction [17]. For example, HPs participating in MCD in average feel that MCD has a positive effect on clinical practice [22]. Therefore, we conducted a study to assess the effect of CES on coercion in psychiatry and on moral skills and attitudes of HPs. Our hypotheses were that with monthly MCD, 1) formal coercive measures in general and seclusion, isolation, and coerced medication in particular become a) less frequent and b) less intense; and 2) HPs show higher moral attentiveness; 3) estimate the intensity of coercion more accurately; 4) exhibit a more negative attitude towards coercion, and 5) disapprove coercion more often than before.

## Methods

### Study design

**Fig. 1:**
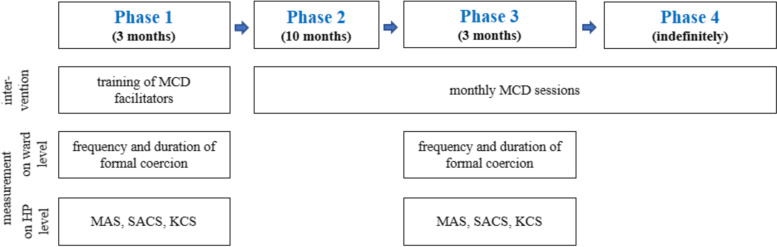
Study design. The study consisted of the intervention (implementation of monthly moral case deliberation (MCD) sessions in phase 2) and pre- and post-measurements (in phase 1 and 3) on ward and healthcare practitioner (HP) level. In phase 4, after completion of the study, MCD sessions are continued on the wards. KCS = Knowledge on Coercion Scale, MAS = Moral Attentiveness Scale, SACS = Staff Attitudes on Coercion Scale

The present study corresponds to a two-center pre-post pilot study (see Fig. 1) that was conducted from June 2019 to September 2020. In phase 1, two experienced and respected HPs from each participating ward who were selected by the chief medical officer were trained as MCD facilitators in a shortened training (three days of training plus self-study and application of the techniques in between) by an MCD expert and pre-implementation measurements were carried out. In phase 2, monthly MCD sessions were conducted by the facilitators who invited all HPs working on the respective ward. In phase 3, MCD sessions continued while post-implementation measurements were performed.

### Setting

The study was conducted on two acute wards in two different psychiatric hospitals in Switzerland (Sanatorium Kilchberg, Canton of Zurich; and Psychiatriezentrum Münsingen, Canton of Bern). The ward in Kilchberg is a closed acute ward specializing in geriatric psychiatry (> 65 years). It has a capacity of 19 beds, one seclusion room and does not apply mechanical restraint. The ward in Münsingen is an open acute ward for adults specializing in psychotic disorders. It can be closed intermittently if necessary and has a capacity of 19 beds and two seclusion rooms.

### Intervention

Monthly *moral case deliberation* (MCD) was implemented on the two participating wards. The MCD sessions in this study addressed concrete, past or anticipated moral challenges related to coercion. Formal coercion was defined as “carrying out a measure in spite of the fact that the person concerned either indicates or has previously indicated – through an expression of wishes or opposition – that he or she does not consent to it “ [2].

A total of 13 MCD sessions were held in Münsingen and 10 in Kilchberg where two sessions had to be canceled due to the COVID-19 pandemic. On average, 7.4 ± 2.6 HPs participated per session in Kilchberg and 8.6 ± 2.2 in Münsingen. In the post-MCD survey, HPs reported to have attended 7.5 ± 5.8 MCD sessions during phase 2. Involvement with CES outside of the study during phase 2 was reported by one participant (3.4%) who self-identified as a member of the hospital’s ethics committee.

### Measures

#### Measures on ward level

The participating wards provided data on staffing and premises, and anonymized data on patients and treatments. The latter is routinely gathered as part of the legally required quality management in Swiss hospitals which is coordinated by the Swiss National Association for Quality Development in Hospitals and Clinics (ANQ). To ensure data quality, the ANQ provides Swiss HPs with information materials and regular trainings. The data provided for this study included the number of patients treated during the respective measurement period, and data on each incident of formal coercion (case ID and main diagnosis of the patient concerned and type, start and – if applicable – end of the coercive measure). Formal coercion is defined by the ANQ as any measure against the – verbally and/or nonverbally expressed – will of the patient concerned, regardless of the intensity of the dissent, previous consent, current decision-making capacity, and the wishes of relatives [23]. Particularly, seclusion is defined as accommodation in a locked room, alone, without the possibility to leave and mechanical restraint is defined as tying to a bed. Interruptions of under two hours, e.g., for personal hygiene or smoking, are disregarded. Coerced medication is defined as administration of one or more drugs, as an injection or perorally, expressly against the patient’s will, with or without restraint. Oral coerced medication includes the explicit threat of coerced injection, should the patient not take the drug orally. Short intervals of restraint to allow for safe administration of coerced medication are not documented separately as incidents of mechanical restraint [23].

#### Measures on health care practitioner level

All staff members of participating wards received an invitation email and two reminders with the link to the online survey (generated with SoSci Survey; [24]) before (phase 1) and after implementation of monthly MCD sessions (phase 3). After clicking on the link, participants were informed that the survey was part of a research project and that they would be asked about their personal values and attitudes, including their personal assessment of several case vignettes. Participants were also assured that the survey was anonymous, and that participation was voluntary. After giving informed consent, participants generated an identification code from personal information (such as the second letter of their mother’s first name) to allow for matching of data from phase 1 and 3. No person-identifying data were collected. HPs provided data on their sociodemographic characteristics, qualification, work experience, previous experience with CES (in phase 1), received CES (in phase 3), and filled out questionnaires.

The *Moral Attentiveness Scale* (MAS) by Reynolds [25] was used in the German translation [24]. The MAS consists of 12 items with a 7-point Likert scale from 1 = “strongly disagree” to 7 = “strongly agree”. A “perceptual” and a “reflective” dimension of moral attentiveness are formed by averaging seven or five of the items, respectively. In addition, it is recommended to sum up the two dimension scores for an overall MAS score [26].

The *Staff Attitude to Coercion Scale* (SACS) by Husum and colleagues [27] was used in the German translation [28]. The SACS consists of 15 items with a 5-point Likert scale from 1 = “disagree strongly” to 5 = “agree strongly”. Items are averaged to yield the scores of the three subscales “coercion as offending attitude” (6 items), “coercion as care and security attitude” (6 items), and “coercion as treatment attitude” (3 items). The SACS has shown acceptable consistency and dimensional validity [29].

In the *Knowledge on Coercion Scale* (KCS) by Jaeger [30], the five levels of coercion by HPs (absence of coercion, persuasion/conviction, leverage, threat, and formal coercion, 4) are represented by three clinical vignettes each. Respondents rate the extent of coercion in the vignettes on 5-point Likert scales from 0 = "no coercion" to 4 = "massive coercion". After calculating the deviation of respondents’ ratings from the default rating, the absolute deviation values are averaged to yield the KCS score with higher scores indicating poorer knowledge of coercion. In addition, for each vignette, respondents indicate dichotomously whether they approved of the portrayed HPs’ conduct. Following Elmer and colleagues [31], we averaged the values from these questions to form the Coercion Attitude (CAT) score.

### Statistical analyses

Data preparation and analysis was conducted using SPSS® version 28.

#### Data preparation

All outliers were checked and verified to ensure that they were true values. By checking participant codes and demographic data, one duplicate dataset was detected and removed from analysis. To further minimize the risk of duplicate data, incomplete questionnaires were removed from the analysis as the participant might have been interrupted and come back to the survey later. Thus, missing data stemmed only from persons not taking part in one of the two surveys at all. In both surveys, there was no difference between persons who participated in only the respective survey versus both surveys regarding age, gender, ward they worked in, work experience, or previous experience with CES (all *p*’s ≥ 0.058). Therefore, the missing data was concluded to be missing completely at random.

The data on coercion were prepared according to the ANQ analysis concept [32] with dichotomous variables indicating whether the corresponding patient had been subjected to any form (or a specific form, respectively) of formal coercion during the measurement period. For each patient subjected to seclusion/mechanical restraint, the intensity of coercion was calculated by summing the duration of all episodes in the measurement period. For each patient subjected to coerced medication, the intensity of coercion was calculated as the absolute frequency of coerced medications during the measurement period.

#### Data analysis

In line with the lack of preexisting data on which to base effect size calculations and the pilot character of this study, hypotheses were tested at the 5% significance level but tendencies (*p* < 0.1) are reported, too, to identify potential effects that might be tested in a follow-up confirmatory study with appropriate statistical power.

Regarding the measures on ward level, since the average stay in the acute wards studied was many times shorter than the interval between the measurement time points, the data on formal coercion from phases 1 and 3 were assumed to stem from different patients and thus be independent. The anonymization of the data precluded verification of this assumption. Considering the clustered nature of the data, we conducted two-level linear mixed effects null models which showed no significant between-cluster variance (all *p*’s ≥ 0.415). We therefore refrained from further multi-level modelling and report analyses aggregated over wards. The absolute frequency of patients subjected coercion versus patients not subjected to it were compared between time points (phase 1 and 3) using the χ^2^ test. Cramér’s *V* was used as effect size and interpreted according to Rea & Parker [33]. Continuous data such as the intensity of seclusion are described as mean ± standard deviation. As Kolmogorov-Smirnoff tests showed significant deviation from the normal distribution, we conducted Mann–Whitney tests for comparisons between measurement time points. As effect size, *r* was calculated and interpreted according to Rosenthal [34].

Regarding the measures on HP level, for calculating descriptive statistics of sociodemographic data and correlations between PVQ-R and SACS subscales, the data from the first participation of each subject was used, that is, the data from phase 1 for all HPs participating in phase 1, and the data from phase 3 for HPs only participating in phase 3. To account for deviations from the normal distribution, 95% confidence intervals for regression coefficients were calculated using unstratified Bias corrected and accelerated (Bca) bootstrapping with 1000 samples. Considering the clustered nature of the data, we conducted three-level linear mixed effects null models which showed no significance between-cluster variance at level 3 (the ward level, all *p*’s ≥ 0.410). We therefore refrained from further modelling this level and report analyses aggregated over wards. To account for missing data, we analyzed the repeated measures (from phase 1 and phase 3) using linear mixed effects models (that is, with time-points clustered in HPs) with diagonal covariance structure (to account for heteroscedasticity), maximum likelihood estimation (to allow for comparisons of model fit), and fixed intercept (as freeing it was not associated with significant improvement in model fit as verified by -2LL χ^2^ tests). Time was specified as a fixed effect.

## Results

### Formal coercion

**Fig. 2:**
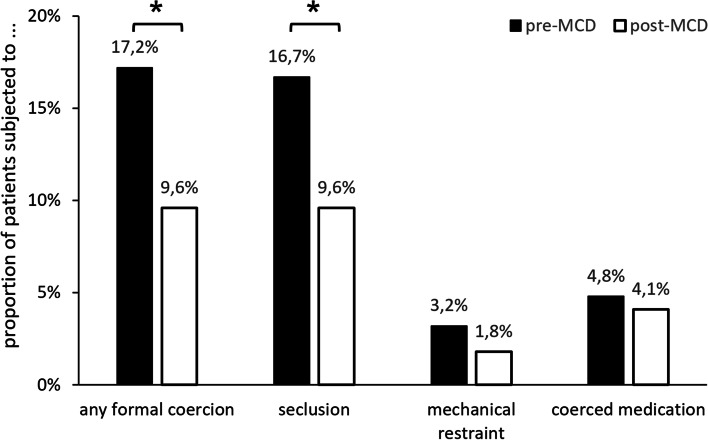
Frequency of formal coercion before and after implementation of MCD. Bar chart showing the frequency of formal coercion, calculated as the proportion of patients subjected to at least one instance of formal coercion (or of a specific type of formal coercion as indicated on the *x* axis) among all patients hospitalized on the participating ward during the respective measurement period (pre-/post-MCD = before/after implementation of monthly moral case deliberation). As some patients were subjected to more than one type of coercion, the sum of the frequencies of the different types of coercion exceeds the frequency of formal coercion in general. * = significant at α ≤ .05

After implementation of MCD, fewer patients were subjected to any form of formal coercion (9.6 vs. 17.2%, χ^2^ (1) = 5.13, *p* = 0.024, Cramér’s *V* = 0.113, see also Fig. 2). This reduction was driven by a smaller proportion of patients being subjected to seclusion (9.6 vs. 16.7%, χ^2^ (1) = 4.50, *p* = 0.034, Cramér’s *V* = 0.105). The proportions of patients subjected to mechanical restraint and coerced medication were numerically lower after implementation of MCD without reaching significance (3.2 vs. 1.8%, χ^2^ (1) = 0.82, *p* = 0.366, Cramér’s *V* = 0.045 and 4.8 vs. 4.1%, χ^2^ (1) = 0.13, *p* = 0.723, Cramér’s *V* = 0.018).

**Fig. 3:**
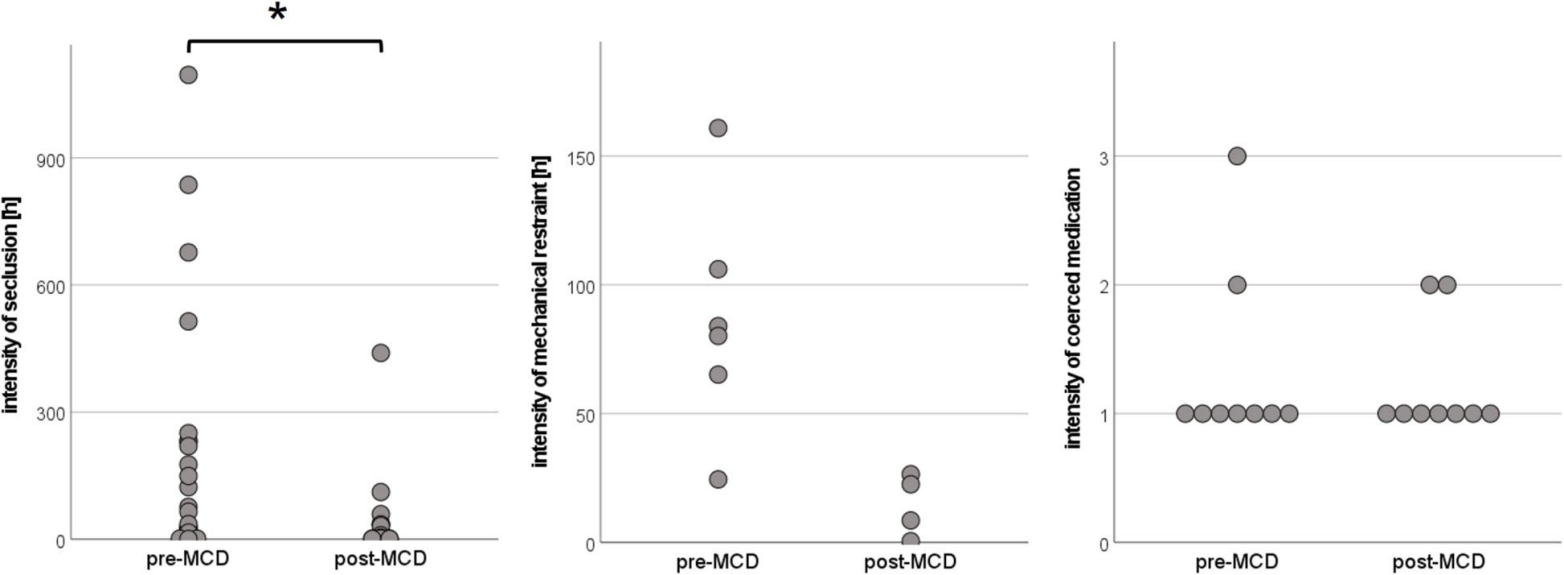
Intensity of formal coercion before and after implementation of MCD. Dot plots of the intensity of types of formal coercion (as indicated on the *y* axis) per patient, showing only data from patients subjected to the respective type of coercion. The intensity of seclusion/mechanical restraint was calculated by summing the duration of individual episodes in the measurement period for each patient concerned. The intensity of coerced medication was calculated as the absolute frequency of coerced medications in the measurement period for each patient concerned (pre-/post-MCD = before/after implementation of monthly moral case deliberation). * = significant at α ≤ .05

Among patients subjected to it, the intensity of mechanical restraint was lower after implementation of MCD (86.8 ± 45.3 vs. 14.5 ± 12.1 h, *U* = 1.00, exact *p* = 0.019, *r* = -0.74, see also Fig. 3). This reduction was driven by a shorter mean duration of restraint episodes (55.2 ± 24.7 vs. 10.1 ± 9.9 h, *U* = 0.00, exact *p* = 0.010, *r* = -0.81), while the absolute frequency of restraint episodes in patients subjected to it was unchanged (1.7 ± 0.8 vs. 1.5 ± 0.6 h, *U* = 11.00, exact *p* = 0.914). Also, there was a trend towards lower intensity of seclusion (156.2 ± 268.8 vs. 39.8 ± 95.2 h, *U* = 241.00, *p* = 0.115, *r* = -0.22). This reduction was driven by a shorter mean duration of seclusion episodes (73.9 ± 102.3 vs. 10.0 ± 12.6 h, *U* = 220.00, *p* = 0.049, *r* = -0.27), while the absolute frequency of episodes in patients subjected to seclusion was unchanged (2.2 ± 2.5 vs. 3.4 ± 6.6 h, *U* = 363.50, *p* = 0.418). Regarding the intensity of coerced medication, there was no difference between before and after implementation of MCD (1.3 ± 0.7 vs. 1.2 ± 0.4 episodes per patient subjected to coerced medication, *U* = 39.50, exact *p* = 0.931).

### Health care practitioners’ attitudes and perceptions

Of the 53 health care practitioners (HPs) that were invited for study participation, 29 participated in the pre-implementation and 28 in the post-implementation measurements (response rates 54.7% and 52.8%, respectively). 11 HPs participated in both measurements. The characteristics of the participating HPs are detailed in Table 1.

**Table 1:** Aggregated sociodemographic characteristics of the participating health care professionals (*N* = 46). CES = clinical ethics support

Age	35.6 ± 13.4 years
Gender	76.1% female
	23.9% male
Education	34.8% primary or secondary
	65.2% tertiary
Area of expertise	79.5% nursing
	2.6% medicine
	7.7% other
	10.3% in training
Work experience (in health care)	11.6 ± 11.2 years
Work experience (in acute psychiatry)	6.9 ± 6.7 years
Work experience (on the current ward)	3.4 ± 4.4 years
Experience with CES prior to the study	28.3%

Approval for coercive measures (as measured with the CAT) showed a trend to be lower after implementation of MCD (*t*(47.4) = 1.83, *p* = 0.073). Exploratory analysis revealed a significant decrease in CAT over time (*F*(1, 38.78) = 6.58, *p* = 0.014) when controlling for the number of MCD sessions the respective HP had attended (*F*(1, 31.0) = 6.59, *p* = 0.015) and the interaction time*number of MCD sessions (*F*(1, 31.0) = 5.44, *p* = 0.026). Pre-post-comparisons for other outcome variables were non-significant (see Supplementary Table).

## Discussion

After implementing monthly moral case deliberation (MCD) on two acute psychiatric wards, the frequency of formal coercion and particularly seclusion was lower (small effect size). Among patients subjected to it, the intensity of mechanical restraint was lower (large effect size), and the intensity of seclusion showed a trend to be lower (small effect size). These effects were driven by respective episodes being shorter. Also, after MCD implementation, healthcare practitioners (HPs) disapproved coercion more often which reached significance only after controlling for the number of MCD sessions attended.

### Efforts to reduce coercion

In the last decades, multiple interventions and strategies have been developed to reduce coercion. Apart from changes in legislation and national policies, and community-based-programs, these are hospital-based initiatives such as *Safewards* and the *Six Core Strategies to Reduce the Use of Seclusion and Restraint®* [35]. In Safewards, HPs prevent triggers for dangerous patient behavior or defuse them, choose not to use formal coercion when it would be counterproductive, and prevent formal coercion from generating further dangerous behavior [36]. The Six Core Strategies include involvement of senior leadership, mandatory and detailed documentation of every seclusion or restraint episode, workforce development (e.g., training in teaching patients emotion regulation), use of prevention tools (e.g., safety plans and environmental changes), involvement of patients and relatives, and debriefing of every seclusion or restraint episode [37].

As elaborated as these programs are, they do not employ or even consider clinical ethics support (CES). However, this study provides preliminary evidence for the effectiveness of CES such as MCD in the reduction of coercion. Rather than being an alternative to existing initiatives, CES is most likely easily integrated with them. For example, HPs employing Safewards may use CES to decide which level of risk of generating further dangerous behavior offsets the potential benefits of coercion. And HPs implementing the Six Core Strategies may call upon CES to deliberate how to deal with a patient refusing the use of prevention tools. Thus, CES such as MCD may be a hitherto underutilized tool to expand and improve existing coercion reduction initiatives.

### Strengths, limitations, and potential explications for null effects

The major strength of this study is the use of formal coercion as an outcome measure that is objective, mandatory, and thus routinely documented in Swiss healthcare system by trained HPs. Another strength of the study is that the coercion was recorded over longer periods of time and at two different clinics.

This study has several limitations. First, as this pilot study was not controlled, the changes in formal coercion and HPs’ attitudes might be due to a factor other than the MCD sessions. For example, the lower approval for coercion among HPs might have been caused by a selection effect (with HPs disapproving of coercion being more likely to support the study by attending MCD sessions and participating in the post-MCD measurement) instead of the MCD sessions themselves. Second, the baseline frequencies of coercion in the participating wards were rather low, especially regarding mechanical restraint and coerced medication. This may have prevented us from detecting a significant reduction (*floor effect*). Third, the quality of the CES might have been lowered by the facilitators receiving only a shortened MCD training due to budgetary restrictions and some MCD sessions having to be postponed or cancelled because of the COVID-19 pandemic. Fourth, this being a pilot study, the sample size was modest both regarding wards and HPs. The low number of participating wards seems the most probable reason for between-ward differences not reaching significance as the variation of coercion use across wards is well documented [8, 9, 11]. The small number of HPs and especially those participating in both measurements might have prevented us from finding more significant changes in HP perceptions and attitudes. Fifth, the HP sample was dominated by nursing staff with only one physician taking part, limiting the generalizability of our findings to other professions. This is problematic as, under Swiss jurisdiction, physicians are the profession ultimately deciding on the use of coercion. Sixth, with our study being conducted in Switzerland, the level of education and training among participating HPs was very high, which limits the generalizability of our findings to other healthcare systems. In less well-funded wards with less highly qualified HPs, other coercion reduction approaches such as expanding HP’s crisis response repertoire [38] might be more effective.

### Future studies

Apart from replicating the positive effects of MCD on coercion in psychiatry in an adequately powered, randomized controlled trial, thus reducing above mentioned limitations, future studies should explore other types of CES such as focused ethical reflection groups [39]. Outcome measures should include not only formal coercion, but also informal coercion [31], perceived coercion [40], and the (mis)match between patients’ individual preferences for types of coercive measures and the type chosen [41]. Furthermore, future studies should evaluate whether CES as add-on to established coercion reduction strategies such as Safewards [36] can help to reduce coercion even further. Elucidating the mechanisms by which MCD in particular or CES in general reduce coercion would help optimizing both CES and coercion reduction programs.

## Conclusion

This study provides preliminary evidence for the effectiveness of moral case deliberations (MCD) in the reduction of the frequency and intensity of formal coercion in acute psychiatric wards. Thus, clinical ethics support (CES) such as MCD might be a hitherto underutilized tool for coercion reduction, complementing existing strategies and programs. Implementing CES may help improve quality of care for persons suffering from mental illness.

## Supplementary Information

**Additional file 1.**

## Data Availability

The datasets used and/or analyzed during the current study are available from the corresponding author on reasonable request.

## Abbreviations

ANQ: Swiss National Association for Quality Development in Hospitals and Clinics
CES: Clinical Ethics Support
HPs: Healthcare professionals
MAS: Moral Attentiveness Scale
MCD: Moral Case Deliberation
SACS: Staff Attitude to Coercion Scale

## Footnotes

1 For present purposes, the term *HPs* was chosen to include all professionals working in mental health care, such as psychiatrists, psychiatric nurses, psychologists, and social workers.
