# Supplementary material for: A two-center pilot study on the effects of clinical ethics support on coercive measures in psychiatry
Source: BMC Psychiatry. 2022 Jun 1;22:370. doi: 10.1186/s12888-022-04024-9 (PMC9156353; doi:10.1186/s12888-022-04024-9)
Supplement: Supplementary file 1 — Additional file 1. [file 12888_2022_4024_MOESM1_ESM.docx]

|  | *M*_EM_ ± *SE* | | *b* ± *SE* | Test statistic of the fixed effect |
| --- | --- | --- | --- | --- |
|  | Pre-MCD | Post-MCD |  |  |
| MAS: perceptual subscale | 4.55 ± 0.21 | 4.56 ± 0.22 | 0.01 ± 0.30 | *F*(1, 57.0) = 0.05, *p* = .962 |
| MAS: reflective subscale | 4.71 ± 0.21 | 4.89 ± 0.23 | 0.18 ± 0.32 | *F*(1, 56.5) = 0.58, *p* = .565 |
| SACS: coercion as offending | 3.31 ± 0.15 | 3.39 ± 0.15 | 0.08 ± 0.21 | *F*(1, 57.0) = 0.40, *p* = .693 |
| SACS: coercion as care & security | 4.13 ± 0.10 | 4.16 ± 0.10 | 0.03 ± 0.14 | *F*(1, 56.9) = 0.24, *p* = .810 |
| SACS: coercion as treatment | 2.21 ± 0.17 | 2.42 ± 0.15 | 0.21 ± 0.23 | *F*(1, 56.3) = 0.91, *p* = .368 |
| KCS | 0.84 ± 0.06 | 0.89 ± 0.07 | 0.06 ± 0.09 | *F*(1, 54.0) = 0.61, *p* = .545 |
| KCS: coercion attitude score | 0.76 ± 0.02 | 0.68 ± 0.04 | 0.08 ± 0.04 | *F*(1, 47.4) = 1.83, *p* = .073 |
| Informal coercion | 2.23 ± 0.14 | 2.19 ± 0.13 | 0.05 ± 0.20 | *F*(1, 55.8) = 0.24, *p* = .809 |

Supplementary Table. Linear mixed effects models of health care practitioners’ attitudes and perceptions of coercion before and after implementation of monthly moral case deliberations (MCD; N = 46)

M_EM_ = estimated marginal means, SE = standard error, b = unstandardized regression coefficient of the main effect of time (reference category: post-MCD measurement), MAS = Moral Attentiveness Scale, SACS: Staff Attitude to Coercion Scale, KCS: Knowledge on Coercion Scale
